# A Self-Referencing Intensity-Based Fiber Optic Sensor with Multipoint Sensing Characteristics

**DOI:** 10.3390/s140712803

**Published:** 2014-07-18

**Authors:** Sang-Jin Choi, Young-Chon Kim, Minho Song, Jae-Kyung Pan

**Affiliations:** 1 Department of Electrical Engineering and Smart Grid Research Center, Chonbuk National University, Jeonbuk 561-756, Korea; E-Mail: sang_jin@jbnu.ac.kr; 2 Department of IT Engineering and Smart Grid Research Center, Chonbuk National University, Jeonbuk 561-756, Korea; E-Mail: yckim@jbnu.ac.kr; 3 Division of Electronic Engineering, Chonbuk National University, Jeonju 561-756, Korea; E-Mail: msong@jbnu.ac.kr

**Keywords:** fiber optic sensor, self-referencing, intensity-based FOS, fiber Bragg grating, multipoint sensing

## Abstract

A self-referencing, intensity-based fiber optic sensor (FOS) is proposed and demonstrated. The theoretical analysis for the proposed design is given, and the validity of the theoretical analysis is confirmed via experiments. We define the measurement parameter, *X*, and the calibration factor, β, to find the transfer function, *H_m_*_,_*_n_*, of the intensity-based FOS head. The self-referencing and multipoint sensing characteristics of the proposed system are validated by showing the measured $H_{m,n}^{2}$ and relative error *versus* the optical power attenuation of the sensor head for four cases: optical source fluctuation, various remote sensing point distances, fiber Bragg gratings (FBGs) with different characteristics, and multiple sensor heads with cascade and/or parallel forms. The power-budget analysis and limitations of the measurement rates are discussed, and the measurement results of fiber-reinforced plastic (FRP) coupon strain using the proposed FOS are given as an actual measurement. The proposed FOS has several benefits, including a self-referencing characteristic, the flexibility to determine FBGs, and a simple structure in terms of the number of devices and measuring procedure.

## 1. Introduction

Over the past 20 years the field of fiber optic sensors (FOS) has been a major user of technology associated with the optoelectronic and fiber optic communications industries [1]. In recent years, FOSs have been used in a wide range of techniques for measurement in areas such as structural health monitoring in renewable energy [2,3], transportation [2,4,5], civil engineering [2,6,7], and the oil and gas industry [2,8–10]. FOS structures can be classified into intensity-based [11–16], spectrally-based [3,4,7,10], and interferometric [5,6,8]. The fiber Bragg gratings (FBG) sensor has been broadly accepted as a structural health monitoring device for fiber-reinforced plastic (FRP) materials, and is applied either by being embedded into or bonded to the structures [17,18].

Among fiber-optic-based sensors, intensity-based FOSs are important for their simplicity and potential low cost, as well as from a historical perspective as they were the first developed. Today they continue to be an attractive choice in many sensing applications due to their ability to measure a wide variety of parameters, their use of inexpensive light sources and simple detection schemes while still benefiting from the intrinsic advantages of photonic sensors: low weight, small size, and electromagnetic immunity. The intensity-based FOS needs a self-referencing characteristic to minimize the influences of long-term aging in source characteristics and to handle short-term fluctuations in optical power loss in the leads to and from the transducer [11–15].

An all-optical technique for multiplexing and self-referencing a number of intensity modulating fiber-optic sensors is described in [11]. Vázquez presents a theoretical and experimental study of radio-frequency ring resonators for referencing and improving the sensitivity of FOS [12]. An improved self-referencing technique using a ring resonator in a new reflection configuration for remote FOS using FBGs is reported in [13]. A self-referencing remote configuration is described as a finite-impulse-response filter in reflective operation using two FBGs and a fiber delay line in [14]. The feasibility of enhancing interrogation automation by working in the virtual domain of remote, intensity-based optical sensors operating in reflective configuration and deployed in radio-frequency wavelength division multiplexing (WDM)-based passive sensor networks is demonstrated in [15]. The interrogation of fiber optic intensity sensors using a combination of the frequency-modulated continuous wave concept with the spectral selective reflectivity of FBGs is demonstrated [16].

In this paper, we propose and implement an intensity-based FOS with self-referencing and cascade and/or parallel multipoint sensing characteristics. In Section 2, the proposed scheme is presented with a theoretical analysis. Section 3 presents an experimental performance validation of the proposed FOS. The power budget analysis and limitations of the measurement rates are also discussed, and the FRP coupon strain under the proposed FOS are given. Finally, we conclude in Section 4.

## 2. Theory and Experimental Setup

**Figure 1.**
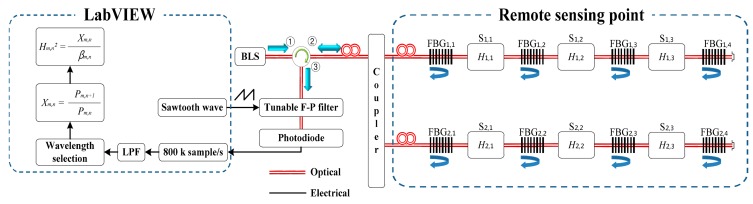
Experimental setup for the proposed self-referencing intensity-based fiber optic sensor.

The proposed intensity-based FOS, which consists of a broadband light source (BLS), fiber optic circulator, optical coupler, FBGs, tunable Fabry-Perot filter (F-P filter), photodiode (PD), and LabVIEW program, is shown in Figure 1. The light from the BLS enters through both the FBG*_m_*_,_*_n_* and the intensity sensor heads, S*_m_*_,_*_n_*, which are located at the remote sensing points via the fiber optic circulator ports, ➀ and ➁. The reflected light from FBG*_m_*_,_*_n_* and FBG*_m_*_,_*_n_*_+1_ return to the PD via an optical coupler, fiber optic circulator, and tunable F-P filter. The reflected light from FBG*_m_*_,_*_n_*_+1_ includes the power modulation of thetravel through the intensity sensor head, S*_m_*_,_*_n_*, which results in a sensitivity enhancement. The intensity sensor head, S*_m_*_,_*_n_*, is assumed to convert the measurands into the corresponding optical intensity. If the resonance wavelength of the filter matches the reflection wavelength of the FBG, the detected power reaches a maximum due to the maximal overlap of the F-P filter passband and the FBG reflection spectrum.

The optical power reflected from FBG*_m_*_,_*_n_* can be expressed as:

(1) $$P_{m,n}=P_{\text{in}}\cdot K_{\text{conn}}\cdot K_{C1-2}\cdot K_{\text{fiber}1}^{2}\cdot K_{\text{coup}}\cdot R_{m,n}\cdot K_{C2-3}\cdot K_{fp},$$

where *P_in_* is the input optical power; *K_conn_* is the loss of the optical connectors; *K_C_*_1−2_ is the loss between fiber optic circulator ports ➀ and ➁; *K_fiber_*_1_ is the loss of the optical fiber between fiber optic circulator port ➁ and FBG*_m_*_,_*_n_*; *K_coup_* is the loss of the optical coupler; *R_m_*_,_*_n_* is the spectral reflectance of FBG*_m_*_,_*_n_*; *K_C_*_2−3_ is the loss between fiber optic circulator ports ➁ and ➂, and *K_fp_* is the loss of the tunable F-P filter. In the same way, the optical power reflected from FBG*_m_*_,_*_n_*_+1_ can be expressed as:

(2) $$P_{m,n+1}=P_{\text{in}}\cdot K_{\text{conn}}\cdot K_{C1-2}\cdot K_{\text{fiber}1}^{2}\cdot K_{\text{coup}}\cdot K_{\text{fiber}2}^{2}\cdot K_{\text{FBGm},n}^{2}\cdot R_{m,n+1}\cdot K_{C2-3}\cdot K_{fp}\cdot H_{m,n}^{2}$$

where *K_fiber_*_2_ is the loss of optical fiber between FBG*_m_*_,_*_n_* and FBG*_m_*_,_*_n_*_+1_; *R_m_*_,_*_n_*_+1_ is the spectral reflectance of FBG*_m_*_,_*_n_*_+1_; *K_FBGm_*_,_*_n_* is the loss of FBG*_m_*_,_*_n_* in the reflective range of the FBG*_m_*_,_*_n_*_+1_ which is determined by the transmission spectrum of the FBG*_m_*_,_*_n_*, and *H_m_*_,_*_n_* is the transfer function of the intensity sensor head, S*_m_*_,_*_n_*. The measurement parameter, *X_m_*_,_*_n_*, is defined using Equations (1) and (2):

(3) $$X_{m,n}=\frac{P_{m,n+1}}{P_{m,n}}=\frac{K_{\text{fiber}2}^{2}\cdot K_{\text{FBGm},n}^{2}\cdot R_{m,n+1}}{R_{m,n}}\cdot H_{m,n}^{2}=\beta_{m,n}\cdot H_{m,n,}^{2}$$

where $\beta_{m,n}=\frac{K_{\text{fiber}2}^{2}\cdot K_{\text{FBGm},n}^{2}\cdot R_{m,n+1}}{R_{m,n}}$, which is equal to *X_m_*_,_*_n_* when the intensity sensor head, S*_m_*_,_*_n_*, has no loss. The value of *H_m_*_,_*_n_* is calculated using *β_m_*_,_*_n_* and *X_m_*_,_*_n_* in Equation (3). The sensitivity of the proposed system can be enhanced because it is possible for the optical signal to travel twice through the intensity sensor, once for each propagating direction of the light.

To validate the self-referencing characteristics of the proposed FOS, we consider that *P_in_* of Equations (1) and (2) changes to *P_in_* + Δ*P_in_*. Although *P_in_* changes to *P_in_*+ Δ*P_in_*, the measurement parameter, *X_m_*_,_*_n_*, in Equation (3) is unchanged, meaning that the proposed system is insensitive to power fluctuation in the input optical source and has self-referencing characteristics. Even if the device parameter has changed, the proposed FOS works correctly only if the calibration factor, β*_m_*_,_*_n_*, is determined initially. The proposed system does not require any other reference FBGs to prepare for the change in device characteristics, which results in a simple and inexpensive structure.

## 3. Measurements

The proposed intensity-based FOS shown in Figure 1 is implemented with a BLS of −16.6 dBm optical power per 0.1 nm bandwidth at 1550 nm, a 1 × 2 50:50 split ratio optical coupler, eight FBGs, six sensor heads, a tunable F-P filter with a wavelength range of 1525–1567 nm, a scan frequency of 3 kHz, and a full width at half maximum (FWHM) of 0.12 nm, and a switchable gain photodiode module. To validate the theoretical analysis with the self-referencing and multipoint sensing characteristics in Section 2, we considered four cases: Optical source fluctuation, various remote sensing point distances, FBGs with different characteristics, and multiple sensor heads with cascade and/or parallel forms. For data acquisition, the proposed intensity-based FOS uses a multifunction DAQ NI-6120 (National Instruments, Austin, TX, USA) LabVIEW program in which the scanning sawtooth wave form is generated for driving a tunable F-P filter. When the driving voltage of the tunable F-P filter is incremented by 0.5 mV, the optical wavelength passing through the tunable F-P filter is decremented by 2 pm. This program operates at a sampling rate of 800 ksamples/s, and a measurement rate of 10 Hz. The power budget analysis and limitations of the measurement rates are discussed, and the FRP coupon strain with the proposed FOS are given as an actual measurement.

### 3.1. Optical Source Fluctuation

**Figure 2.**
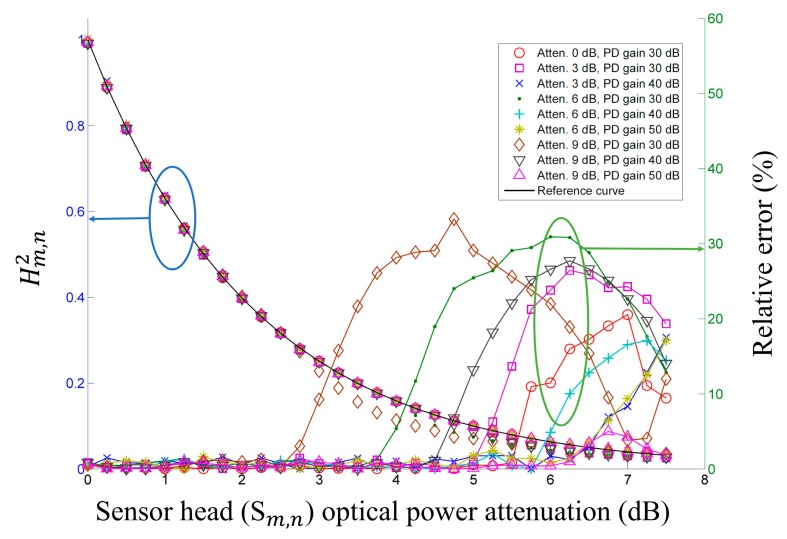
Measured $H_{m,n}^{2}$ (left) and relative error (right) *versus* sensor head optical power attenuation with various source optical power attenuations and PD gains.

To test the self-referencing characteristic, we consider that the optical source is attenuated by an optical attenuator inserted between the BLS and the fiber optic circulator, as shown in Figure 1. This power attenuation, up to 9 dB, is similar to the input optical power variation that might be expected if optical power of the last sensor head fluctuated in multiple sensor heads with cascade forms. This experiment uses FBG*_m_*_,_*_n_* with a central wavelength(λ_c_) of 1549.45 nm, a FWHM (Δλ) of 0.48 nm, a reflectance (*R_m_*_,_*_n_*) of 77.66%, FBG*_m_*_,_*_n_*_+1_ with a λ_c_ of 1547.58 nm, a Δλ of 0.48 nm, and an *R_m_*_,_*_n_*_+1_ of 77.09%. *β_m_*_,_*_n_* is calculated as 0.3763 using Equation (3). Figure 2 shows the measurement results of $H_{m,n,}^{2}$ the relative error for the input optical power with attenuations of 0 dB, 3 dB, 6 dB, and 9 dB, and a sensor head optical power variation of 0 to 7.5 dB, with step increments of 0.25 dB via a bi-directional optical level attenuator (OLA-55m, JDSU, Milpitas, CA, USA). The measured results in Figure 2 are close to the reference curve, and we can see that the proposed FOS has a self-referencing characteristic that minimizes the influences of long-term aging in the source characteristics and short-term fluctuations in optical power loss. We can see that the relative errors are less than 2% when the measurement parameter *X_m_*_,_*_n_* is larger than 0.04, which corresponds to a PD input optical power of approximately −40 dBm. When measurement parameter *X_m_*_,_*_n_* is less than 0.04, the measurement errors increase rapidly. Figure 2 shows that when the operating range is defined as the sensor head optical power attenuation range in which the relative error is less than 2%, the operating range increases as the source optical power attenuation decreases and the PD gain increases.

### 3.2. Remote Sensing Point Distances

**Figure 3.**
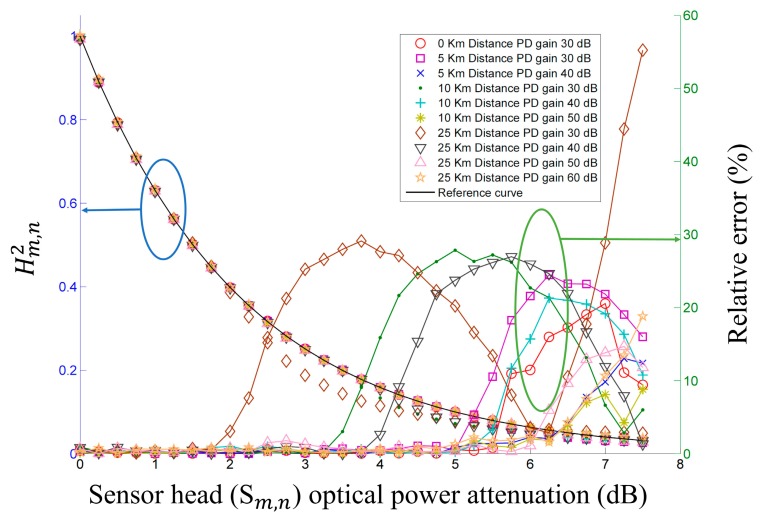
Measured $H_{m,n}^{2}$ (left) and relative error (right) *versus* sensor head optical power attenuation with various remote sensing point distances and PD gains.

To test the proposed FOS's performance according to the remote sensing point distance, we used optical fiber roll lengths of 5 km, 10 km, and 25 km between the fiber optic circulator and the optical coupler shown in Figure 1. The FBG specifications are the same as those in the above self-referencing characteristic test. Figure 3 shows the measured results of $H_{m,n}^{2}$ and the relative error for the various remote sensing point distances with different PD gains. Used optical fiber has an attenuation of 0.194 dB/km and a polarization mode dispersion of 0.06 ps/km at 1550 nm. As shown in Figure 3, the operating range increases as the optical fiber length decreases and the PD gain increases.

### 3.3. FBGs with Different Characteristics

**Figure 4.**
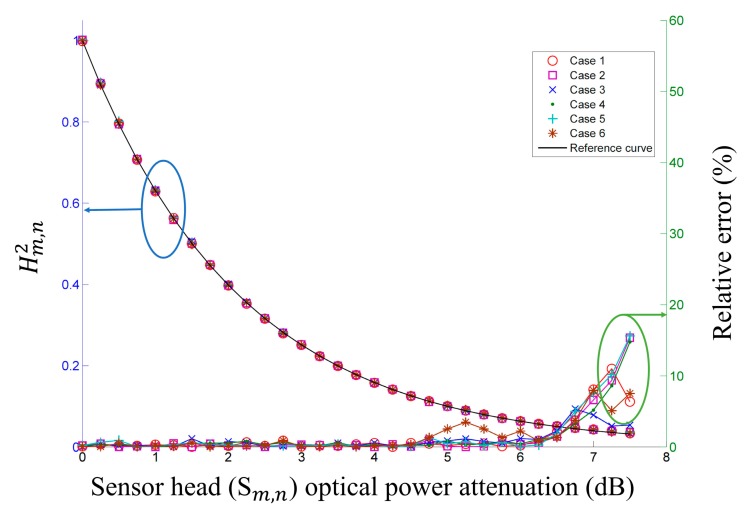
Measured $H_{m,n}^{2}$ (left) and relative error (right) *versus* sensor head optical power attenuation of six cases from Table 1.

To test the proposed FOS performance according to the FBGs characteristics used, we used six different FBG pairs, as shown in Table 1. As shown in Figure 4, the measured $H_{m,n}^{2}$ for FBGs with different characteristics, such as those in Table 1, are close to the reference curve. When the PD gain is 40 dB under the specifications of cases 1–6, the operating ranges of the sensor heads are up to 6.25 dB, 6.50 dB, 6.25 dB, 6.50 dB, 6.50 dB, and 6.50 dB, respectively. These results show that FBGs with arbitrary characteristics can be used to implement the proposed FOS.

### 3.4. Multipoint Sensing Characteristic

**Figure 5.**
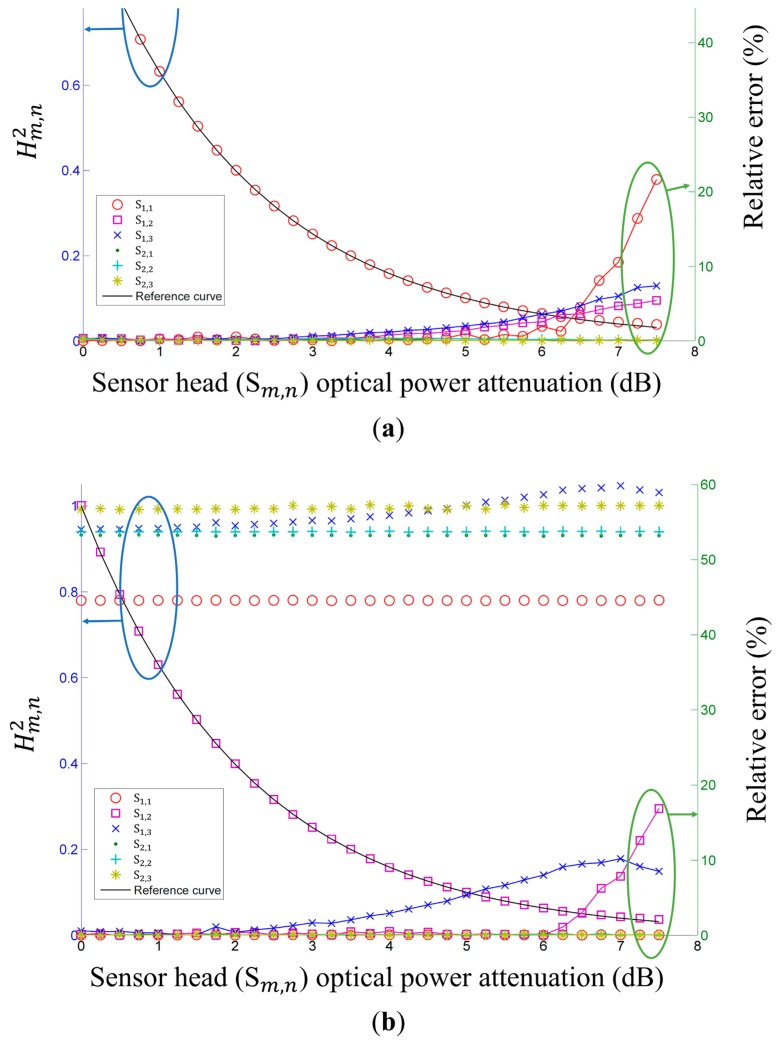

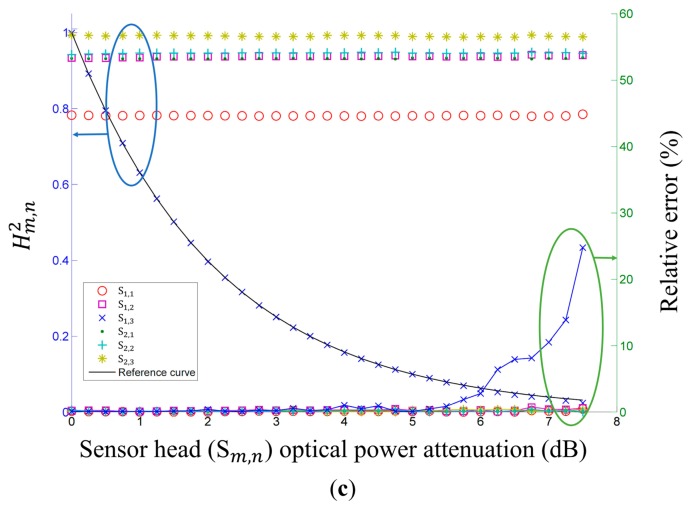
Measured $H_{m,n}^{2}$ (left) and relative error (right) *versus* sensor head optical power attenuation with multiple sensor heads. (**a**) S_1,1_ with optical power variation and other sensor heads with no optical power variation; (**b**) S_1,2_ with optical power variation and other sensor heads with no optical power variation; (**c**) S_1,3_ with optical power variation and other sensor heads with no optical power variation.

To test the multipoint sensing characteristic of the proposed FOS, we implemented the proposed FOS with eight FBGs with central wavelengths (λ_c_) of 1544 to 1558 nm with a step of 2 nm, a FWHM (Δλ) of 0.48 nm, and a reflectance (R*_m_*_,_*_n_*) of 77%∼80%. We measured the $H_{m,n}^{2}$ and relative error with six cascade and/or parallel multipoint sensors, as shown in Figure 5. Figure 5a–c shows the measured $H_{m,n}^{2}$ and relative error *versus* the sensor head optical power variation according to the multiple sensor heads: (a) S_1,1_ with optical power variation and other sensor heads with no optical power variation; (b) S_1,2_ with optical power variation and other sensor heads with no optical power variation; and (c) S_1,3_ with optical power variation and other sensor heads with no optical power variation. S_1,1_, S_1,2_, and S_1,3_ with sensor head optical power variation are in good agreement with the reference curve. The other measured $H_{m,n}^{2}$ for fixed sensor heads are in relatively good agreement with the applied sensor heads.

### 3.5. Power Budget and Measurement Rates

For a given set of components and system requirements, we carry out a power budget analysis to determine whether the operating range of the proposed FOS meets the calculated power margin. The proposed FOS loss budget simply considers the total optical power loss *P_T_* that is allowed between the BLS and the PD shown in Figure 1, and allocates this loss primarily to components loss, connector loss, optical fiber attenuation, and power variation of the sensor head. Thus, if *P_in_* is the optical power emerging from the BLS, and if *P_R_* is the PD sensitivity, then:

(4) $$\begin{array}{ll} P_{T} & =P_{\text{in}}-P_{R}\\ & =K_{\text{conn}}+K_{C1-2}+2K_{\text{fiber}1}+K_{\text{coup}}+2K_{\text{fiber}2}+2K_{\text{FBGm},n}+R_{m,n+1}+K_{C2-3}+K_{\text{fp}}+\text{power variation of the sensor head} \end{array}$$

In our experiments, *P_in_* = −16.6 dBm, the PD gain and sensitivities (*P_R_*) are 40 dB and −40 dBm, 40 dB and −44 dBm, 50 dB and −48 dBm, and 60 dB and −52 dBm, which result in the allowed loss of 23.4 dB, 27.4 dB and 31.4 dB, respectively. The losses of the components used in the experiments are shown in Table 2. The following three experiments were considered to calculate the power budget and to validate the experimental results regarding the operating range of the FOS:

(a) Experiment 1: the FOS with a source optical power attenuation of 9 dB and PD gains of 30, 40, and 50 dB, respectively. (Figure 2)
(b) Experiment 2: the FOS with a remote sensing point distance of 25 km and PD gains of 30, 40, 50, and 60 dB, respectively. (Figure 3)
(c) Experiment 3: the FOS with *S*_1,1_, *S*_1,2_, and *S*_1,3_ with optical power variation and other sensor heads with no optical power variation. (Figure 5)

We can see that the measured operating range of the FOS from the experimental results of Figures 2, 3, and 5 meets the calculated power margin in Table 2. The largest difference between the measured operating range of the FOS and the calculated power margin appears in experiment 2 with a PD gain of 60 dB, due to the low optical power of the sensor head output. This corresponds to an *X_m_*_,_*_n_* of less than 0.04.

**Figure 6.**
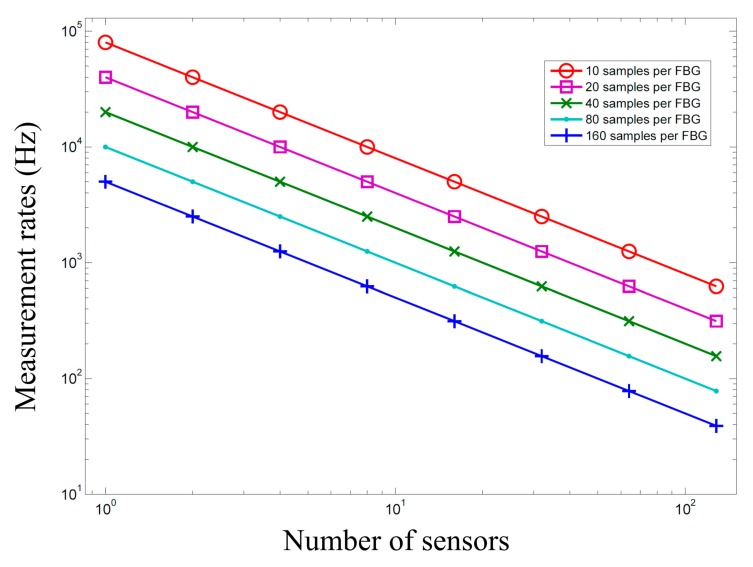
The measurement rates according to the number of sensors with different samples per FBG, measuring the FBG reflected waveforms.

Figure 6 shows calculated measurement rates according to sampling number, in order to measure the FBG reflected waveform acquired by the Gaussian curve fitting method, and the number of sensors in Figure 1. The measurement rates were calculated using the DAQ NI 6120 (16-Bit) with a sampling rate of 800 ksamples/s. The measurement rates decreased with increasing sample per FBG as well as with increasing numbers of sensors.

### 3.6. Measurement of FRP Coupon Strain

**Figure 7.**
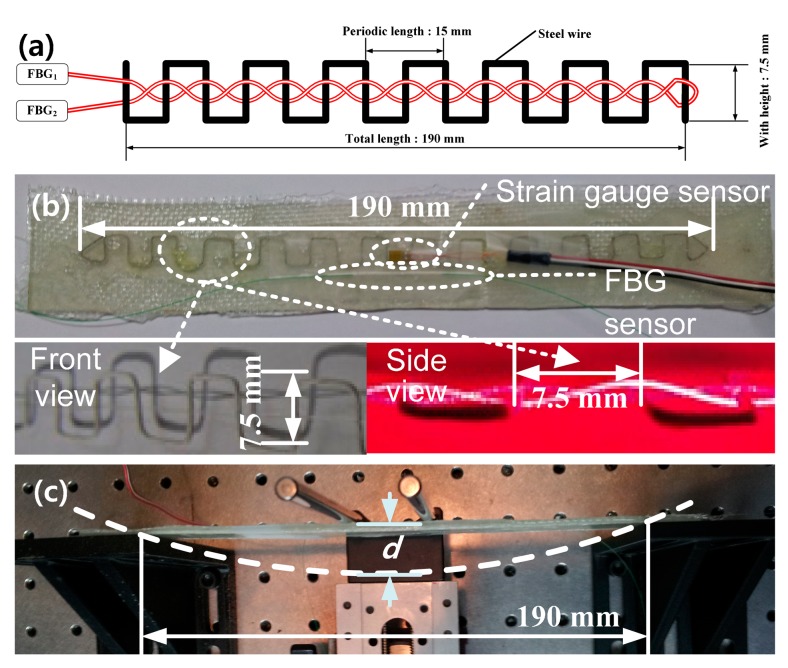
A schematic and experimental setup for the measurement of FRP coupon strain. (**a**) Schematic of the proposed intensity-based FOS head; (**b**) three types of strain sensors bonded on the FRP surface; (**c**) experimental setup to measure FRP coupon.

To apply the proposed FOS to an actual measurement, we implemented a sensor head with FRP material, and measured the FRP coupon strain with the proposed FOS. The intensity-based FOS head shown in Figure 7a is composed of a rectangular pulse-train-shaped steel wire with a height of 7.5 mm, periodic length of 15 mm, and total length of 190 mm. The optical fiber and wire are set to cross each other 25 times. The wire is bonded on an FRP coupon with a length of 220 mm via epoxy resin. The implemented FOS head shows an insertion loss of approximately 1 dB and an operating range extending to 25 dB.

Figure 7b shows three types of strain sensors bonded on the FRP surface: the intensity-based FOS, FBG sensor (I-MON 512E, Ibsen, Farum, Denmark), and the strain gauge sensor (D4 Data Acquisition, Micro-Measurement, Wendell, NC, USA). The experimental setup to measure the FRP coupon strain is shown in Figure 7c, where *d* is the displacement of the FRP coupon.

**Figure 8.**
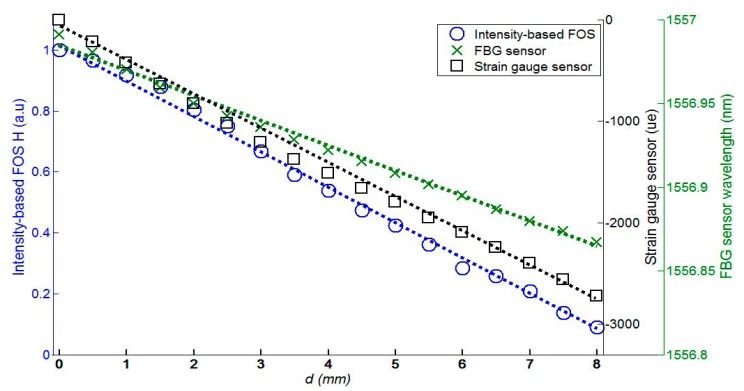
Measured data of FRP coupon strain with three types of sensors (*d*: displacement in Figure 7c).

We measured the FRP coupon strain simultaneously using three types of strain sensors: the proposed FOS head, FBG sensor, and strain gauge sensor, as shown in Figure 7b. The measured FRP coupon strain data is shown in Figure 8 according to the displacement, *d*, in Figure 7c with three types of sensors. While the displacement, *d*, varies from 0 to 8 mm with increments of 0.5 mm via a micro-stage, the transfer function, *H*, of the proposed FOS head, wavelength of the FBG sensor, and strain of the strain gauge sensor vary from 1 to 0.0745265, from 1556.98 to 1556.86 nm, and from 0 to 2640.117 με, respectively. Dotted lines in Figure 8 represent the linear fit of the measured data. The average relative error and the maximum relative error of the proposed FOS head, FBG sensor, and strain gauge sensor are 2.95% and 7.39%, 3.55% and 10.46%, and 3.39% and 7.66%, respectively. These results show that the self-referencing, intensity-based FOS interrogator with the proposed FOS head displays good performance when measuring FRP coupon strain.

## 4. Conclusions

A novel intensity-based FOS, with self-referencing and multipoint sensing characteristics, relatively simple structure, and low cost has been proposed and demonstrated. The self-referencing and multipoint sensing characteristics of the proposed system are validated by showing the measured $H_{m,n}^{2}$ and relative error *versus* the sensor head optical power attenuation for the optical source fluctuation, various remote sensing point distances, FBGs with different characteristics, and six sensor heads with cascade and/or parallel forms. The power budget analysis and limitations of the measurement rates are discussed, and the measurement results of the FRP coupon strain with the proposed FOS are given as an actual measurement. We have confirmed that the operating range for the proposed FOS increases as the input optical power increases, the optical fiber length decreases, and the PD gain increases. The FRP strain data using the proposed scheme was compared with those of the FBG and strain gauge sensors. The average relative error and maximum relative error of the proposed FOS, FBG sensor, and strain gauge sensor are 2.95% and 7.39%, 3.55% and 10.46%, and 3.39% and 7.66%, respectively.

## Figures and Tables

**Table 1.** Specifications for FBG*_m_*_,_*_n_* and FBG*_m_*_,_*_n_*_+1_ used in the experiments.

**Cases**	**FBG***_m_***_,_***_n_*			**FBG*_m_*_,_*_n_*_+1_**			***β****_m_***_,_***_n_*
	
	**λ_c_(nm)**	**Δλ (nm)**	***R****_m_***_,_***_n_***(%)**	**λ_c_(nm)**	**Δλ (nm)**	***R****_m_***_,_***_n_***_+1_(%)**	
Case1	1549.45	0.48	77.66	1556.69	0.18	80.00	0.5017
Case2	1556.69	0.18	80.00	1549.45	0.48	77.66	0.2932
Case3	1554.15	0.89	99.59	1549.45	0.48	77.66	0.1860
Case4	1549.45	0.48	77.66	1554.15	0.89	99.59	0.5124
Case5	1556.69	0.18	80.00	1554.15	0.89	99.59	0.4348
Case6	1554.15	0.89	99.59	1556.69	0.18	80.00	0.3888

**Table 2.** Spreadsheet for calculating the proposed FOS power budget.

**Component/Loss Parameter**		**Experiment 1**			**Experiment 2**				**Experiment 3**		
									**(a)**	**(b)**	**(c)**
BLS output (dBm)		−16.6			−16.6				−16.6		
PD gain (dB)/sensitivity (dBm)		30/−40	40/−44	50/−48	30/−40	40/−44	50/−48	60/−52	40/−44	40/−44	40/−44
Allowed loss (dB)		23.4	27.4	31.4	23.4	27.4	31.4	35.4	27.4	27.4	27.4
Loss (dB)	Attenuator	9.0									
	Connectors (*K_conn_*)	1.2			1.2				1.8		
	Circulator (*K_C_*_1−2_, *K_C_*_2−3_)	1.7			1.7				1.7		
	Fiber (*K_fiber_*_1_, *K_fiber_*_2_)	0.6			10.3				0.6		
	Coupler (*K_coup_*)								3.2		
	*FBG_m_*_,_*_n_* transmission spectrum (*K_FBGm_*_,_*_n_*)	0.2			0.2				0.2		
	*FBG_m_*_,_*_n_*_+1_ spectral reflectance (*R_m_*_,_*_n_*_+1_)	0.6			0.6				0.6		
	F-P filter (*K_fp_*)	4.5			4.5				4.5		
	Previous sensor head (*H_m_*_,_*_n_*)									1.1	1.6
	Total loss	17.8			18.5				12.6	13.7	14.2
Power margin (dB)		5.6	9.6	13.6	4.9	8.9	12.9	16.9	14.8	13.7	13.2
Measured operating range of the FOS (dB) (Bidirection)		5.0	9.0	12.5	3.5	7.5	12.0	12.5	12.5	12.5	11.5

## References

[1] Yin S.S., Ruffin P.B., Yu F.T.S. (2008). Fiber Optic Sensors.

[2] López-Higuera J.M., Rodriguez Cobo L., Quintela Incera A., Cobo A. (2011). Fiber Optic Sensors in Structural Health Monitoring. IEEE J. Light. Technol..

[3] Schroeder K., Ecke W., Apitz J., Lembke E., Lenschow G. (2006). A fibre Bragg grating sensor system monitors operational load in a wind turbine rotor blade. Meas. Sci. Technol..

[4] Friebele E.J., Askins C.G., Bosse A.B., Kersey A.D., Patrick H.J., Pogue W.R., Putnam M.A., Simon W.R., Tasker F.A., Vincent W.S. (1999). Optical fiber sensors for spacecraft applications. Smart Mater. Struct..

[5] Gaizka D., Marlene K., Michael L., de Idurre S.O., Hans P., Joseba Z., Carmen V. (2009). Use of a Novel Fiber Optical Strain Sensor for Monitoring the Vertical Deflection of an Aircraft Flap. IEEE Sens. J..

[6] Bolster M., Deblois R., French C., Phipps A., Sebasky J., Western K. Structural Health Monitoring System for the new I-35W St Anthony Falls Bridge.

[7] López-Higuera J.M., Misas C.J., Incera A.Q., Cuenca J.E. (2005). Fiber optic civil structure monitoring system. Opt. Eng..

[8] Huang S.-C., Lin W.-W., Tsai M.-T., Chen M.-H. (2007). Fiber optic in-line distributed sensor for detection and localization of the pipeline leaks. Sens. Actuators A Phys..

[9] Zhong Z.Y., Zhi X.L., Yi W.J. Oil Well Real-time Monitoring With Downhole Permanent FBG Sensor Network.

[10] Nellena P.M., Maurona P., Franka A., Sennhausera U., Bohnertb K., Pequignotb P., Bodorb P., Brändleb H. (2003). Reliability of fiber Bragg grating based sensors for downhole applications. Sens. Actuators A Phys..

[11] Spillman W.B., Lord J.R. (1987). Self-referencing multiplexing technique for fiber-optic intensity sensors. J. Light. Technol..

[12] Vázquez C., Montalvo J., Lallana P.C. (2005). Radio-frequency ring resonators for self-referencing fibre-optic intensity sensors. Opt. Eng. Lett..

[13] Vázquez C., Montalvo J., Montero D.S., Pena J.M.S. (2006). Self-referencing fiber-optic intensity sensors using ring resonators and fiber Bragg gratings. IEEE Photonics Technol. Lett..

[14] Montalvo J., Frazão O., Santos J.L., Vázquez C., Baptista J.M. (2009). Radio-frequency self-referencing technique with enhanced sensitivity for coarse WDM fiber optic intensity sensors. J. Light. Technol..

[15] Montero D.S., Vázquez C. (2013). Remote Interrogation of WDM Fiber-Optic Intensity Sensors Deploying Delay Lines in the Virtual Domain. Sensors.

[16] Perez-Herrera R.A., Pereira D.A., Frazão O., Castro Ferreira J.M., Santos J.L., Araújo F.M., Ferreira L.A., Baptista J.M., Lopez-Amo M. (2011). Optimization of the frequency-modulated continuous wave technique for referencing and multiplexing intensity-based fiber optic sensors measurement. Measurement..

[17] Frieden J., Cugnoni J., Botsis J., Gmür T. (2012). Low energy impact damage monitoring of composites using dynamic strain signals from FBG sensors—Part II: Damage identification. Compos. Struct..

[18] Lau K.T., Yuan L., Zhou L.M., Wu J., Woo C.H. (2001). Strain monitoring in FRP laminates and concrete beams using FBG sensors. Compos. Struct..

